# MiR-1307 promotes ovarian cancer cell chemoresistance by targeting the ING5 expression

**DOI:** 10.1186/s13048-016-0301-4

**Published:** 2017-01-11

**Authors:** Wen-Ting Chen, Yu-Jia Yang, Zhen-Dong Zhang, Qiang An, Na Li, Wei Liu, Bing Yang

**Affiliations:** Affiliated hospital of Zunyi Medical College, Zunyi, Guizhou 563000 People’s Republic of China

**Keywords:** miR-1307, Ovarian cancer, Cell chemoresistance, ING5

## Abstract

**Background:**

We aimed to investigate the function of miR-1307 in chemoresistance and to explore its chemoresistance mechanism in ovarian cancer.

**Methods:**

IC50 determination was used to test the chemoresistance profling in ovarian cancer cells. QRT-PCR or western blot was used to validate the expression level of miR-1307 and candidate gene or protein. Colony formation assay and FITC-labeled enhanced Annexin V immunofluorescence were used to compare cell proliferation and apoptosis ability, respectively. The potential target gene and its biological function of miRNA-1307 were also analyzed. Bioinformatics and Luciferase Reporter Gene Assay were conducted to validate the regulation of miRNA-1307 on the ING5 expression. Xenografts assay was used to demonstrate the inhibiting effect of miR-1307 ASO and Taxol therapy against ovarian cancer in vivo.

**Results:**

MiR-1307 was over-expressed in chemoresistant ovarian cancer cell line A2780/Taxol, and over-expression or loss of miR-1307 promoted or inhabited chemoresistance. And we also found that the over-expression of miR-1307 promoted proliferation and inhibited apoptosis in ovarian cancer cells. Besides, we demonstrated that ING5 was a direct target of miR-1307 and miR-1307 down-regulated the ING5 expression in ovarian cancer cells. Additionally, we showed that ING5 inhibited cell proliferation, promoted cell apoptosis and inhabited chemoresistance reversely. Furthermore, the up-regulated ability of cell apoptosis and down-regulated ability of chemoresistance following the loss of miR-1307 was reversed by adding ING5 siRNA in vitro. Finally, we proved the inhibiting effect of miR-1307 ASO and Taxol therapy by increasing the ING5 expression against ovarian cancer through xenografts assay in vivo.

**Conclusion:**

Our results suggested that miR-1307 could promote ovarian cancer chemoresistance by targeting the ING5 expression and miR-1307 might serve as a therapeutic target for ovarian cancer.

## Background

Ovarian cancer is a malignancy with with the fifth mortality in female malignant tumors and the highest mortality rate in gynecological cancers, of which epithelial ovarian carcinoma (EOC) is the most common pathologic type accounting for 85–90%. It is estimated that there will be 22,280 Americans diagnosed with ovarian cancer in 2016, and 14,240 of them will die from the disease [1]. The high mortality rate of ovarian cancer is associated with the difficulties of early detection, because most patients are not diagnosed until late stage (stage III or IV) in their disease [2]. Besides, for the patients of ovarian cancer, the majority experience relapse within 2 years [3]. Chemotherapy plays an important role in the therapy for ovarian cancer, but chemoresistance during chemotherapy makes treatment particularly challenging. The chemoresistance has been one of the main reasons for the high mortality of ovarian cancer [4]. Therefore, it is urgent to discover new treatment strategies for reducing the occurrence of chemoresistance to help improve prognosis.

As a class of small non-coding RNA molecules, miRNAs are endogenously expressed, single-stranded and 19–25 nucleotides long [2, 5, 6]. MiRNAs, as transcriptional repressors, regulate gene expression by directly binding the 3’ untranslated region of their target miRNAs [2, 5, 6]. Numerous studies had proved that miRNAs are involved in regulation of almost all cellular processes including proliferation and apoptosis [2, 5–7]. Recently, miRNAs have been reported to either promote carcinogenesis by inhibiting tumor suppressors or suppress tumor development by acting as down-regulate oncogenes in ovarian cancer: downregulated miRNAs (including let-7a/b/d/f, miR-31, 34abc, 92a, 99b, 125b, 127, 152, 155 and 199a), and over-expressed oncogenic miRNAs (such as miR-18a, 20a, 21, 23a/b, 29a, 92, 93, 126, 141, 199a-3p, 200b/c and 429) [2, 8–12]. Moreover, about 27 dysregulated miRNAs have been linked to chemo-resistance to taxanes or platinum compounds in ovarian cancer [13]. Over-expression of miR-27a and miR-514 or loss of let-7i/let-7e have been related to resistance to taxanes and/or platinum [13, 14]. MiR-93 and MiR-214 can promote cisplatin resistance by targeting PTEN/AKT [9, 15]. MiR-376c can promote cisplatin resistance by targeting ALK7 [9]. MiR-214 can promote paclitaxel resistance by targeting BCL10 and caspase-7, and miR-433 or miR-182 can promote paclitaxel resistance by targeting MAD2 or PDCD4 [9]. MiR-141 can promote platinum resistance by targeting KEAP1 [9]. Two studies have reported that miRNA is involved in the development of chemoresistance in ovarian cancer by inhibiting pro-apoptotic signal pathway [4, 8]. Additionally, up-regulation of miR-300 can inhibit cellular apoptosis through TGF-β, resulting in chemoresistance enhancement in ovarian cancer cells [16]. Particularly, it has been reported that miRNA-1307 is over-expressed in chemoresistant ovarian cancer tissues compared to the chemosensitive counterparts, indicating that miR-1307 is associated with the chemoresistance in ovarian cancer [7]. However, up to now, the functional study of miR-1307 has been limited, and the chemoresistance mechanism of miR-1307 in ovarian cancer is still unclear.

In the present study, we evaluated the miR-1307 expression in chemoresistant ovarian cancer cell line A2780/Taxol and the function of miR-1307 for chemoresistance in various kinds of ovarian cancer cells. We also performed a systematic analysis on miR-1307 for its role in ovarian cancer chemoresistance and a preliminary analysis on the mechanism. Finally, our results indicated miR-1307 could promote ovarian cancer chemoresistance by reducing the ING5 expression in vitro and in vivo. Thus, miR-1307 might serve as a therapeutic target for ovarian cancer.

## Methods

### Cell culture

Human ovarian cancer cell line A2780, SKOV3 and paclitaxel-resistant A2780 (A2780/Taxol) were obtained from the the Committee of Type Culture Collection of the Chinese Academy of Sciences (Shanghai, China). The SKOV3 and A2780/Taxol cells were maintained in RPMI-1640 medium, while the A2780 cellswere maintained in Dulbecco’s modified Eagle’s medium (DMEM). Ten percent fetal bovine serum (FBS), 100 units · mL^−1^ penicillin and 100 μg · mL^−1^ streptomycin were added in the above. All cells were incubated at 37 °C in a humidified atmosphere of 95% air and 5% CO_2_.

### Chemoresistance profling (IC50 determination) [17, 18]

Cells were seeded in triplicate in 96-well plates at the density of 5 × 10^3^/well and treated differently or not in the various kinds of ovarian cancer cells for 72 h. Cell survival was then measured by a thiazolyl blue tetrazolium bromide (MTT, 490 nm reading)-based cell proliferation assay. Both the linear regression parameters and the IC50 (the concentration of drug required for 50% of cells to be killed) with the no-drug control as the reference were calculated. The relative chemoresistance was presented as the fold for each of the cell line over the lowest IC50.

### RNA analysis

Total RNA was isolated from the cells at the logarithmic phase using TRIzol reagent (Tiangen Biotech Co., Ltd., Beijing, China). For mRNA analysis, cDNA primed by oligo-dT was generated using a PrimeScript RT reagent kit (Tiangen Biotech Co., Ltd., Beijing, China), and the mRNA levels of the genes were quantifed by duplex-QRT-PCR analysis using TaqMan probes with a different fuorescence for the β-actin (provided by Shing Gene, Shanghai, China) and a FTC-3000P PCR instrument (Funglyn Biotech Inc, Canada). PCR cycle conditions are: 95 °C for 5 min, followed by 40 cycles of 95 °C 10s, 60 °C 20s, 72 °C 20s, and 78 °C 20s. Using the 2-∆∆Ct method, gene expression was normalized to β-actin and then compared between groups. The sequences of the primers and probes used for the QRT-PCR analysis were as follows:

- miR-1307 F: 5’-AACTCGGCGTGGC -3’,
- miR-1307 R: 5’-GAGCAGGCTGGAGAA-3’.
- U6 was used as internal control in RT-PCR:
- U6 F: 5’-GCTTCGGCAGCACATATACTAAAAT-3’,
- U6 R: 5’-CGCTTCACGAATTTGCGTGTCAT-3’.
- ING5 F: 5’- TCCAGAACGCCTACAGCAAG -3’,
- ING5 R: 5’- TGCCCTCCATCTTGTCCTTC -3’,
- ING5 probe: 5’- CY5-CGACAAAGTGCAGCTG GCCATGC -3’.

Experiment was performed independently for three times.

### Reagents for the transient transfection assays

The miR-1307 mimics, miR-1307 ASO, siRNA and the scramble sequence control were supplied by Guangzhou Ribobio (Guangzhou, China). The transfection was used by Lipofectamine-2000 in accordance with the manufacturer’s guidelines (Invitrogen). The final concentration of antisense oligodeoxynucleotide (ASO) con and miR-1307 ASO was 100 nM. Untreated cells were designated as the blank control group.

The siRNA sequences used for ING5 interference in this study was as follows:

- 5’CCAUGUACUUGGAGCACUA dTdT 3’,
- 3’dTdT GGUACAUGAACCUCGUGAU 5’.

### Cell proliferation

Cell proliferation ability was compared by colony formation assays. 6-well plates were used to seed cell suspensions, 300 cells per well. After incubated, cells were fixed in methyl hydrate for 10 min. Then the colonies were stained and they were counted by using an optical microscope.

### Western blot

A quantity of 30 ug of lysates per sample was separated by SDS–PAGE using 10% polyacrylamide gels and transferred to PVDF membrane which was subsequently incubated with the antibody (see the below) for 4 °C overnight, and corresponding were immunodetected by incubation with HRP (horseradish peroxidase)-linked related secondary antibody using an ECL detection kit (Pierce Biotechnology). The antibodies used in this article were below: rabbit monoclonal Ki67 antibody (sc-15,402, 1:1000 dilution), mouse monoclonal PCNA antibody (sc-25,280, 1:1000 dilution), mouse monoclonal caspase-3 antibody (sc-65,496, 1:1000 dilution), mouse monoclonal caspase-7 antibody (sc-81,655, 1:1000 dilution), rabbit monoclonal ING5 antibody (10665-1-AP, 1:1000 dilution), mouse monoclonal β-actin antibody (sc-8432, 1:2500 dilution), the secondary antibodies preparation was either anti-rabbit (1:5000) or anti-mouse (1:5000).

### Apoptosis analysis by FITC immunofluorescence

Cells were harvested and rinsed with phosphate-buffered saline (PBS) twice. Then, 5 μl of fuorescein isothiocyanate (FITC)-labeled enhanced Annexin V and 5 μl (20 μg/ml) of propidium iodide were added to the 100 μl cell suspension. Following incubation in the dark for 15 min at room temperature, the samples were diluted with 400 μl PBS. Then dropped the cell suspension on the slide after dyeing, and covered it with a cover glass cell for detection by using fluorescence microscope. Annexin V-FITC showed the green fluorescence, and DAPI showed the blue fluorescence. The results were analyzed according to the manufacturer’s instructions. The experiments were performed independently three times, and a representative result was shown herein.

### Analysis of miRNA target genes and gene ontology analysis

Target Scan, miRanda and Diana microT-CDS were used to analyze the potential target genes for miR-1307. GOstat was used for gene enrichment analysis. The DAVID database was used for signal transduction enrichment Analysis.

### Dual-luciferase reporter assay [19]

5 × 10^4^ cells per well in 12-well plates were cultured without antibiotics overnight and then transfected with cloned ING5 wild-type 3’-UTR target sequence and mutant 3’-UTR by using Lipofectamine 2000 (Invitrogen). After 48 h, cells were washed with PBS, subjected to lysis, and their luciferase activities measured by using a dual luciferase assay kit (Promega). The results were normalized against Renella Luciferase. Each reporter plasmid was transfected at least three times. The wild sequence for ING5: 3’ UTR: AGCUGGCCCUCGACGCCCGGACC, while mutant sequence was 3’UTR: ACCAGCCGCUCGACGCCCGGACC. Both of them were designed and purchased from Shanghai Genechem Co., Ltd (Shanghai, China).

### Xenografts assays in vivo

The animal study was carried out in accordance with the guidelines approved by the Animal Experimentation Ethics Committee of affiliated hospital of Zunyi medical college. The protocol was approved by the committee, all surgery was performed under sodium pentobarbital anesthesia, and all efforts were made to minimize suffering. Athymic Balb/c nude mice (aged 5 weeks) were provided by Slac Laboratory Animal Co. Ltd. (Shanghai, China), and the mice were housed in a pathogen-free animal facility and randomly assigned to the control or experimental group (six mice per group) [20]. 2 × 10^6^ A2780 cells were suspended in 0.1 mL of serum-free RPMI 1640 was injected into the right subaxillary of each mouse [20]. And the mice were divided into 5 groups which were treated differently on the day 14 (the tumor volume reached about 50–100 mm^3^): The mice were injected with MOCK (transfected with vector), Taxol, ASO con, miR-1307 ASO, or miR-1307 ASO + Taxol once every 2 days (8 times in all) [17]. The final concentration of MOCK, ASO con and miR-1307 ASO was 100 nM intratumorally injected into A2780 cells every time, respectively (0.01 mol). And taxol was injected 10 mg/kg for the mice intraperitoneally. The tumors were measured by vernier caliper on the day 14, 17, 21, 23, 26, and 29. The following formula was used for calculation of tumor volume: Tumor volume (mm^3^) = tumor length (mm) × tumor width (mm) × tumor width (mm)/2. 29 days after inoculation, mice were killed, and the final volume of tumor tissues was determined.

### Statistical analysis

The experiments’ results in vitro and in vivo were depicted as mean ± SD. Student’s two-sided *t*-test was used to compare values of test and control samples. All calculations were performed with the SPSS 18.0 and the level of significance was set to *P* < 0.05.

## Results

### MiR-1307 was over-expressed in chemoresistant ovarian cancer cell line A2780/taxol, and over-expression or loss of miR-1307 promoted or inhabited chemoresistance

**Fig. 1:**
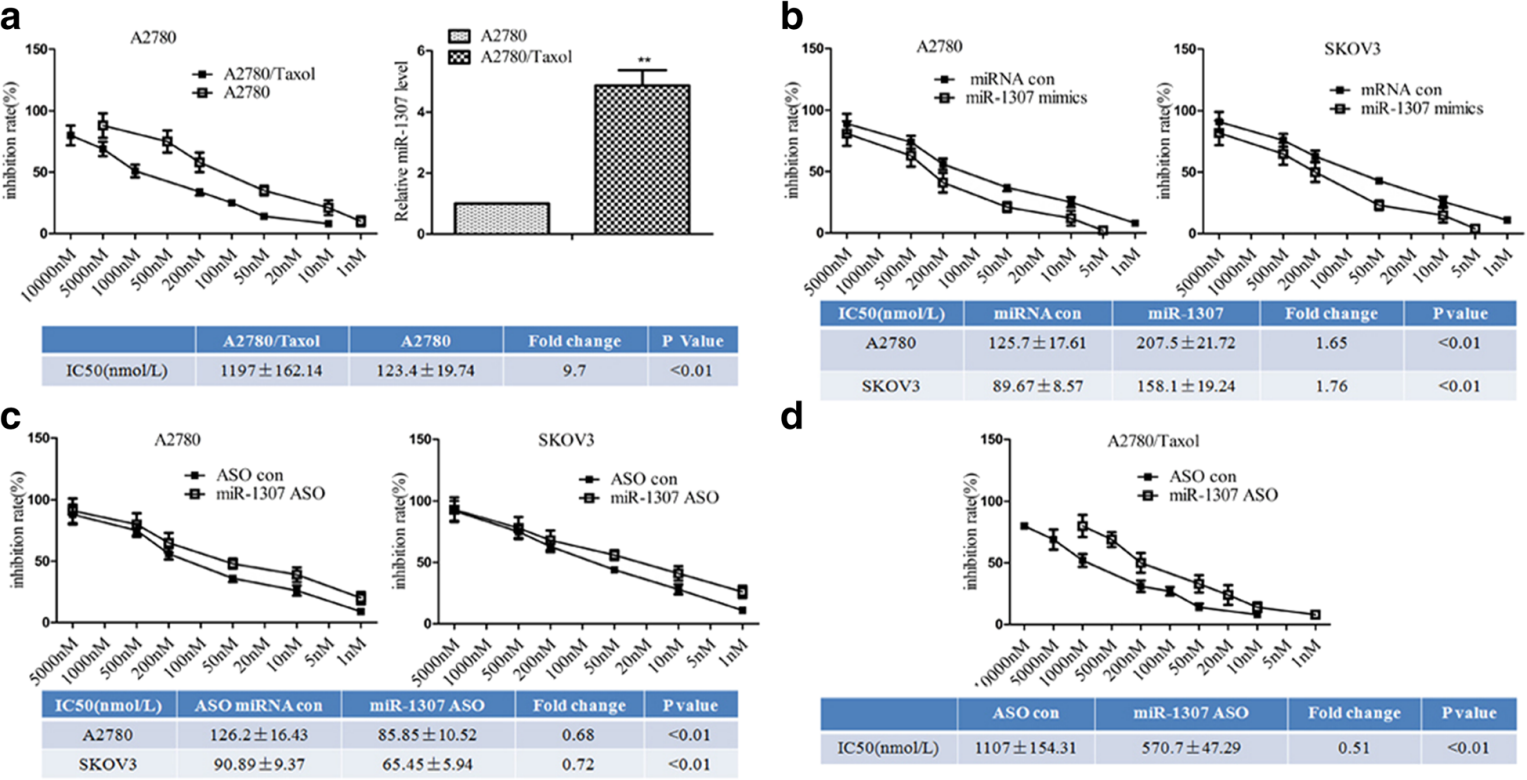
The over-expression and function of miR-1307 in chemoresistant ovarian cancer cells. **a** Chemoresistance of A2780 and A2780/Taxol cells. The relative cell survival rates over mock treatment was calculated and plotted. Diagram line about different concentrations of drugs and inhibition rate, the IC50 difference between A2780 and A2780/Taxol cells and the miR-1307 expression were shown (the same below). The IC50 and the relative fold were presented in table (the same below). The IC50 in the chemoresistant A2780/Taxol cells was much bigger than that in the A2780 cells (*P* < 0.01). QRT-PCR results indicated miR-1307 was over-expressed in A2780/Taxol cells compared to A2780 cells (*P* < 0.01). **b** Chemoresistance of miR-1307 mimics transfection and control group in the A2780 or SKOV3 cells. The results of IC50 determination revealed that the IC50 was bigger in the miR-1307 mimics group than that in the miRNA control group in the A2780 or SKOV3 cells (*P* < 0.01, respectively). **c** Chemoresistance of miR-1307 ASO transfection and control group in the A2780 or SKOV3 cells. The results of IC50 determination revealed that the IC50 was smaller in the miR-1307 ASO group than that in the ASO control group in the A2780 or SKOV3 cells (*P* < 0.01, respectively). **d** Chemoresistance of miR-1307 ASO transfection and control group in the A2780/Taxol cells. And the results of IC50 determination revealed that the IC50 was smaller than that in the ASO control group in the A2780/Taxol cells (*P* < 0.01). At least three independent experiments were conducted. Data from three experimental determinations and bars indicate the SD. Data are expressed as the mean ± standard deviation (** *P* < 0.01)

To prove the characteristics of chemoresistant ovarian cancer cells, we showed the IC50 difference between the A2780 and A2780/Taxol cells: IC50 in the chemoresistant A2780/Taxol cells was much bigger than that in the A2780 cells (*P* < 0.01, Fig. 1a). To determine if miR-1307 had a role in chemoresistant ovarian cancer cells, we performed QRT-PCR to detect the miR-1307 expression in the A2780 and A2780/Taxol cells. Our results indicated that miR-1307 was over-expressed in chemoresistant ovarian cancer cell A2780/Taxol, and perhaps miR-1307 promoted cell chemoresistance in cell A2780/Taxol compared to cell A2780 (*P* < 0.01, Fig. 1a). To further investigate the apoptosis effect of miR-1307 in ovarian cancer cells, we transferred miR-1307 mimics or ASO to increase or decrease miR-1307 expression both into the A2780 or SKOV3 cells. The results of IC50 determination revealed that increased or decreased miR-1307 expression could promote or inhibit chemoresistance (IC50 bigger or smaller than the control, *P* < 0.01, respectively, Fig. 1b and c). To demonstrate the therapy effect on the chemoresistant ovarian cancer cells, we transferred miR-1307 ASO into the A2780/Taxol cells. And the results of IC50 determination revealed that decreased miR-1307 expression could inhabit chemoresistance (IC50 smaller than the control, *P* < 0.01, Fig. 1d).

To sum up, these results indicated that miR-1307 was over-expressed in chemoresistant ovarian cancer cell line A2780/Taxol, and miR-1307 promoted chemoresistance.

### Over-expression of miR-1307 inhibited cell apoptosis and promoted cell proliferation in ovarian cancer cells

**Fig. 2:**
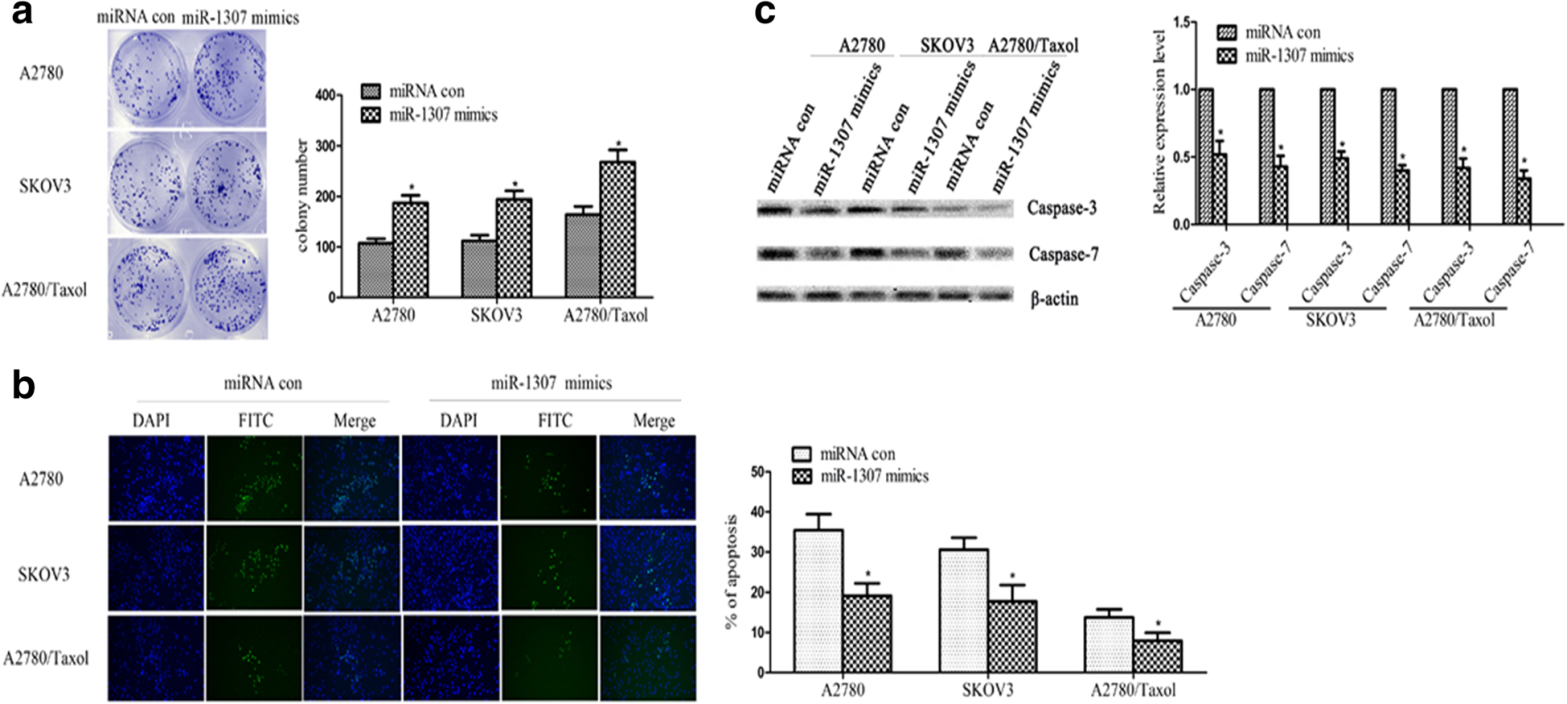
Influence of miR-1307 on the biological behaviors of ovarian cancer cell lines. MiR-1307 mimics or control was transferred into three ovarian cancer cells (A2780, SKOV3 and A2780/Taxol). **a** Colony formation. Cells were cultured in 6-well plates and analyzed by colony formation assay. After 10 days, cells were stained, photographed (*left*) and counted (*right*). The results of colony formation assay showed that capacity for colony formation was significantly enhanced in miR-1307 mimics transfection cells compared to control in the A2780 or SKOV3 cells (*P* < 0.05, respectively). **b** The results of annexin V-FITC immunofluorescence assay. Cells were stained, photographed (left) and counted (right). The results of annexin V-FITC immunofluorescence assay showed significantly more cells of FITC in miR-1307 mimics transfection cells compared to control in the A2780 or SKOV3 cells (*P* < 0.05, respectively). **c** The expression level of protein caspase-3 and caspase-7 by using western blot in different groups. The expression level of protein caspase-3 or caspase-7 in miR-1307 mimics transfection cells was higher than that in the control in the A2780 or SKOV3 cells (*P* < 0.05, respectively). At least three independent experiments were conducted. Data from three experimental determinations and bars indicate the SD. Data are expressed as the mean ± standard deviation (* *P* < 0.05)

Moreover, to performe a systematic analysis on miR-1307 for its role in ovarian cancer chemoresistance, we transferred miR-1307 mimics into three ovarian cancer cells (A2780, SKOV3 and A2780/Taxol) and analyzed the effect on the proliferation and apoptosis compared to the control. The results of colony formation assay showed that capacity for colony formation was significantly enhanced in miR-1307 mimics transfection cells compared to control (*P* < 0.05, respectively, Fig. 2a). Besides, the results of annexin V-FITC immunofluorescence assay showed significantly more cells of FITC in miR-1307 mimics transfection cells compared to control (*P* < 0.05, respectively, Fig. 2b). Additionally, to prove the above results and analyze the mechanism of apoptosis, we used western blot method to detect the protein of caspase-3 and caspase-7, which were used to be genetic markers of apoptosis. As shown in Fig. 2c, the expression level of protein caspase-3 and caspase-7 in miR-1307 mimics transfection cells was higher than that in the controls, respectively (*P* < 0.05).

In a word, these results indicated that over-expression of miR-1307, which was associated with chemoresistance, inhibited cell apoptosis and promoted cell proliferation in ovarian cancer cells.

### ING5 was a direct target of miR-1307 and miR-1307 down-regulated the ING5 expression in ovarian cancer cells

**Fig. 3:**
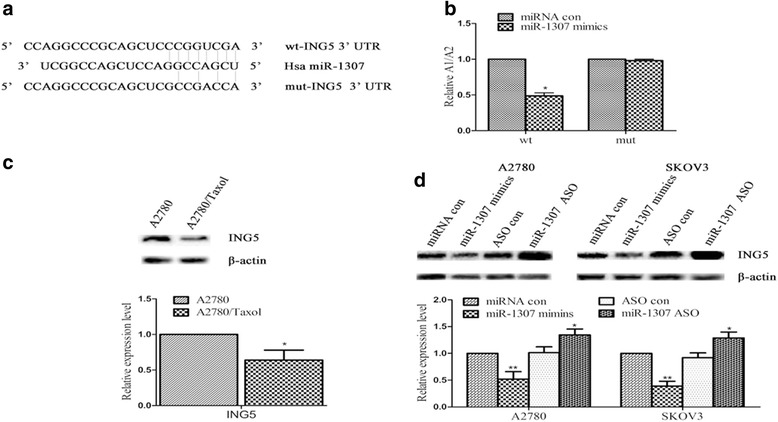
MiR-1307 down-regulated the direct target ING5 expression in vitro. **a** Bioinformatics results. The 3’-untranslated region (3’-UTR) of ING5 contains a potential miRNA-binding site for miR-1307. We inserted the ING5 UTR (1–1037 bp) with either a wild-type or mutant miR-1307 target sequence downstream of the firefly lucierase gene into the pGL3-control vector (Promega) to create the pGL3-ING5 UTR WT or the pGL3-ING5 UTR Mut construct, respectively. **b** Luciferase reporter assay results. The pGL3-ING5 UTR WT, pGL3-ING5 UTR Mut and pGL3 constructs were individually transfected into A2780 cells. MiR-1307 significantly decreased the relative luciferase activity of the wild-type ING5 3’UTR compared with the mutant ING5 3’UTR. **c** The ING5 protein expression level following different miR-1307 treatment in A2780/Taxol and A2780 cells by using western blot. The results showed the ING5 protein expression level was lower in A2780/Taxol cells than that in A2780 cells (*P* < 0.05). **d** The ING5 protein expression level following different miR-1307 treatment in ovarian cancer cells by using western blot. The ING5 protein expression level was lower treated with miR-1307 mimics than that treated with miRNA con in the A2780 or SKOV3 cells (*P* < 0.05, respectively). Moreover, the ING5 protein expression level was higher treated with miR-1307 ASO than that treated with ASO con in the A2780 or SKOV3 cells (*P* < 0.05, respectively). Results are representative of at least three separate experiments. Data are expressed as the mean ± standard deviation. Data from three experimental determinations and bars indicate the SD (*P < 0.05, **P < 0.01)

To identify the downstream target genes of the miR-1307, we performed chip analysis and found that ING5 is a target gene of miR-1307 (unpublished data, not shown). Incidentally, ING5 was on the list of miR-1307 targets suggested by Target Scan software (http://www.targetscan.org/). In addition, to demonstrate the regulatory effect on miR-1307 and downstream gene ING5, we inserted the ING5 UTR (1–1037 bp) with either a wild-type or mutant miR-1307 target sequence downstream of the firefly lucierase gene into the pGL3-control vector (Promega) to create the pGL3-ING5 UTR WT or the pGL3-ING5 UTR Mut construct, respectively (Fig. 3a). The pGL3-ING5 UTR WT, pGL3-ING5 UTR Mut and pGL3 constructs were individually transfected into A2780 cells. Then we performed luciferase reporter assays with the wild-type or mutant 3’UTR of miR-1307. Our results demonstrated that miR-1307 significantly decreased the relative (A1/A2: pGL3-ING5 UTR WT or pGL3-ING5 UTR Mut construct group / pGL3 construct group) luciferase activity of the wild-type miR-1307 3’UTR compared to the mutant miR-1307 3’UTR, indicating that miR-1307 might directly bind to the 3’UTR of ING5 (Fig. 3b, *P* < 0.05). In conclusion, the ING5 gene is a direct post-transcriptional target of miR-1307.

To further validate the relationship between miR-1307 and ING5 in ovarian cancer cells, we detected the level of ING5 protein expression following different miR-1307 treatment in various kinds of ovarian cancer cells by western blot. The results showed the ING5 protein expression level was lower in A2780/Taxol cells than that in A2780 cells (Fig. 3c, *P* < 0.05). From the above results (Fig. 1a), we knew that the miR-1307 expression level was lower in A2780/Taxol cells than that in A2780 cells (*P* < 0.01). Moreover, our results showed the ING5 protein expression level was lower treated with miR-1307 mimics than that treated with miRNA con in the A2780 or SKOV3 cells, respectively (Fig. 3d, *P* < 0.01). Furthermore, our results showed the ING5 protein expression level was higher treated with miR-1307 ASO than that treated with ASO con in the A2780 or SKOV3 cells, respectively (Fig. 3d, *P* < 0.05). That was to say, over-expression or loss of miR-1307 could down- or up-regulated the expression level of protein ING5. Taken together, miR-1307 could down-regulate the ING5 expression in ovarian cancer cells.

### The loss of ING5 expression promoted cell proliferation, inhibited cell apoptosis, and promoted chemoresistance in ovarian cancer cells

**Fig. 4:**
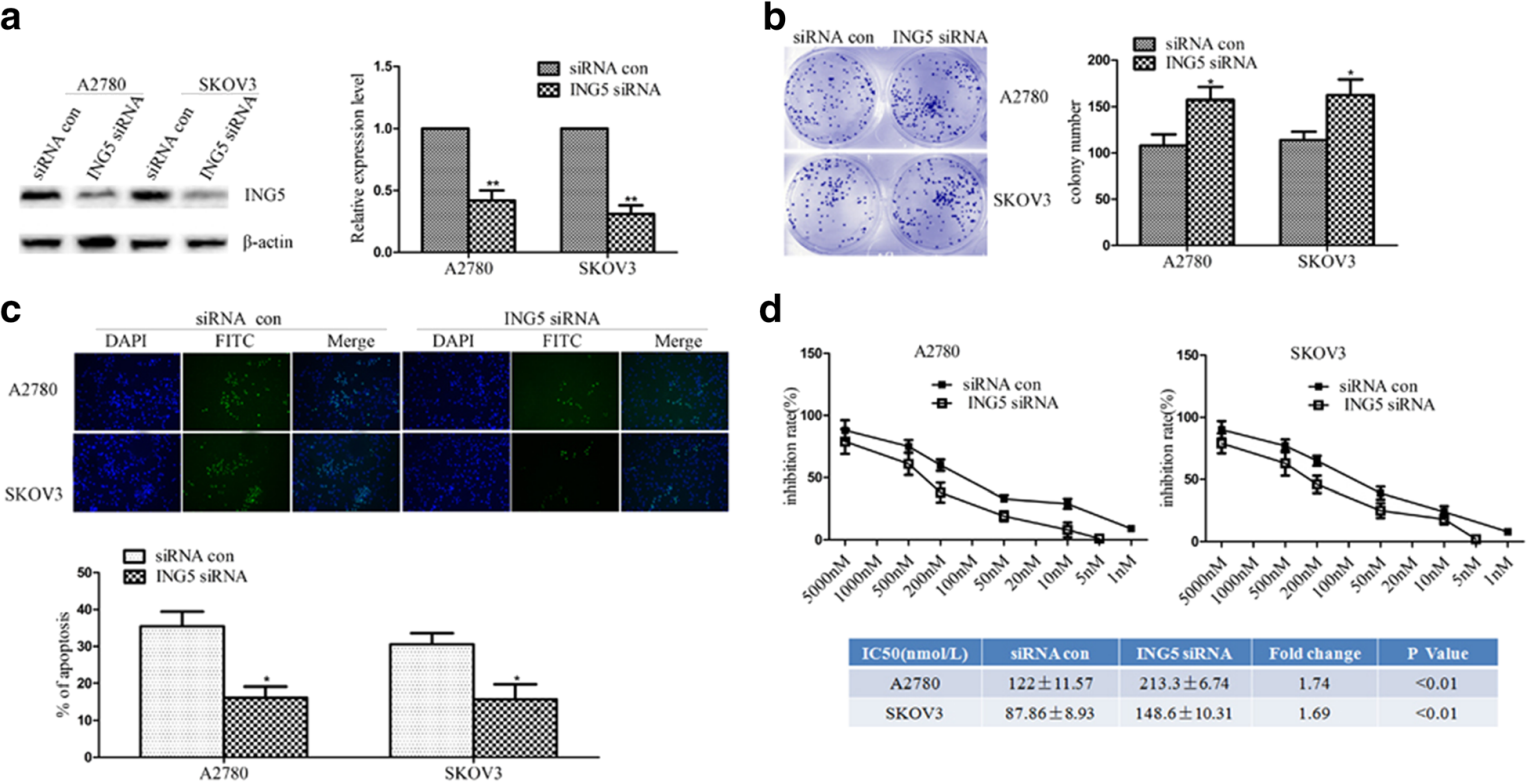
Influence of ING5 on the biological behaviors of ovarian cancer cell lines. **a** ING5 siRNA was used to reduce the expression of protein ING5 in A2780 and SKOV3 cells by using western blot. The expression of ING5 protein in A2780 or SKOV3 cells was reduced in the ING5 siRNA group compared to the siRNA control group (*P* < 0.01, respectively). **b** Colony formation. Cells were cultured in 6-well plates and analyzed by colony formation assay. After 10 days, cells were stained, photographed (left) and counted (right). The results of colony formation assay or annexin V-FITC immunofluorescence assay showed that capacity for colony formation was significantly enhanced compared to the control in A2780 or SKOV3 cells, respectively (*P* < 0.05). **c** The results of annexin V-FITC immunofluorescence assay. Cells were stained, photographed (up) and counted (down). The results of annexin V-FITC immunofluorescence assay showed that significantly more cells of FITC compared with control in A2780 or SKOV3 cells (*P* < 0.05, respectively). **d** Chemoresistance profiling of ING5 siRNA transfection and control group in the A2780 and SKOV3 cells. Diagram line about different concentrations of drugs and inhibition rate, and IC50 difference between ING5 siRNA transfection and control group in the A2780 and SKOV3 cells. The results of IC50 showed that significantly bigger IC50 compared with control in A2780 or SKOV3 cells (*P* < 0.05, respectively). Results are representative of at least three separate experiments. Data are expressed as the mean ± standard deviation. Data from three experimental determinations and bars indicate the SD (*P < 0.05, **P < 0.01)

To investigate the influence of ING5 on the biological behaviors of ovarian cancer cells, ING5 siRNA was used to reduce the expression of ING5 in A2780 and SKOV3 cells: as shown in Fig. 4a, the expression of ING5 in A2780 or SKOV3 cells was reduced, respectively (*P* < 0.01). The results of colony formation assay or annexin V-FITC immunofluorescence assay showed that capacity for colony formation was significantly enhanced compared to the control in A2780 or SKOV3 cells, respectively (*P* < 0.05, Fig. 4b). The results of annexin V-FITC immunofluorescence assay or IC50 showed that significantly more cells of FITC or bigger IC50 compared with control in A2780 or SKOV3 cells (*P* < 0.05, respectively, Fig. 4c and d). As a whole, the results indicated that loss of ING5 expression promoted cell proliferation, inhibited cell apoptosis, and promoted chemoresistance in ovarian cancer cells.

### MiR-1307 promoted the development the chemoresistance of ovarian cancer by targeting ING5 in vitro

**Fig. 5:**
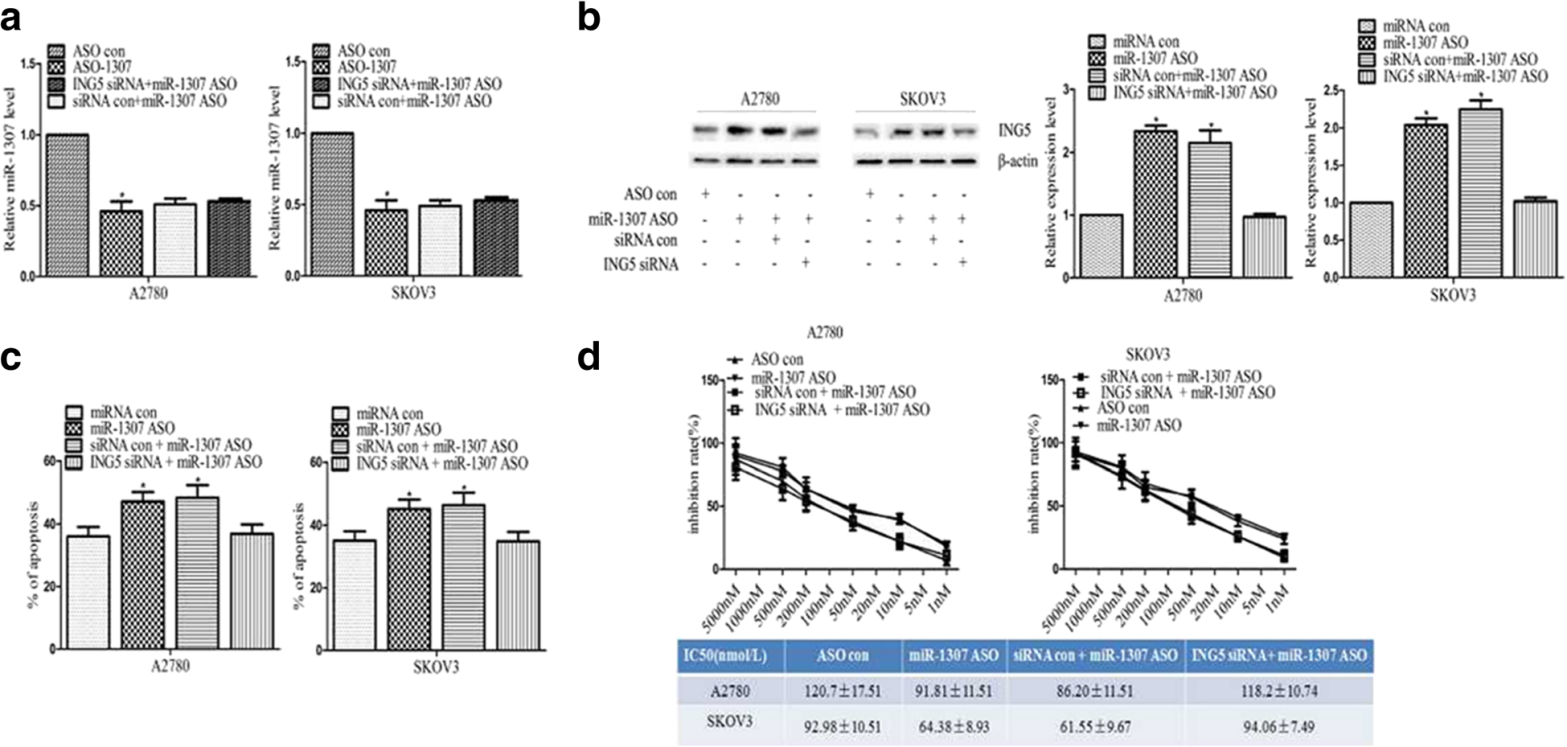
The changed apoptosis or chemoresistance following miR-1307 loss was rescued by ING5 down-regulation. **a** The miR-1307 expression by using QRT-PCR in A2780 or SKOV3 cells in the differently treated groups: The miR-1307 expression was decreased in the ASO-1307 following the loss of miR-1307 compared to the ASO con group (*P* < 0.05, respectively). Besides, the decreased miR-1307 expression was not statistically different between the ING5 siRNA + miR-1307 ASO group or in the siRNA con + miR-1307 ASO group following the loss of miR-1307 in A2780 or SKOV3 cells (*P* > 0.05, respectively). **b** The ING5 protein by using western blot in A2780 and SKOV3 cells in the differently treated groups: The ING5 expression was increased in the ASO-1307 or in the siRNA con + miR-1307 ASO group following the loss of miR-1307 in A2780 or SKOV3 cells (*P* < 0.05, respectively). Meanwhile, the increased ING5 expression was not increased by adding ING5 siRNA following the loss of miR-1307 in A2780 or SKOV3 cells (*P* < 0.05, respectively). **c** The results of apoptosis rate in the different groups in A2780 or SKOV3 cells: The results of annexin V-FITC immunofluorescence assay showed that the apoptosis rate increased in the ASO-1307 or in the siRNA con + miR-1307 ASO group following the loss of miR-1307 in A2780 or SKOV3 cells (*P* < 0.05, respectively). However, the increased cells apoptosis rate following the loss of miR-1307 could be rescued (not increased) by down-regulation of ING5 in A2780 or SKOV3 cells (*P* < 0.05, respectively). **d** The results of chemoresistance profiling in the different groups in A2780 and SKOV3 cells: The chemoresistance was decreased in the ASO-1307 or in the siRNA con + miR-1307 ASO group following the loss of miR-1307 (*P* < 0.01, respectively). However, the decreased chemoresistance following the loss of miR-1307 could be rescued (not increased) by down-regulation of ING5 in A2780 or SKOV3 cells (*P* < 0.05, respectively). (*P < 0.05)

The results described above pointed out a possibility that miR-1307 promoted ovarian cancer cells progression by down-regulating ING5. To further demonstrate this, we reversed the cell apoptosis and chemoresistance abilities by adding ING5 siRNA following the loss of miR-1307 at the same time (Fig. 5). As shown in Fig. 5a, the miR-1307 expression was decreased in the ASO-1307 following the loss of miR-1307 compared to the ASO con group in A2780 or SKOV3 cells (*P* < 0.05, respectively). And the decreased miR-1307 expression was not statistically different between the ING5 siRNA + miR-1307 ASO group or in the siRNA con + miR-1307 ASO group following the loss of miR-1307 in A2780 or SKOV3 cells (*P* > 0.05, respectively). The results indicated that the loss of miR-1307 was not increased by adding ING5 siRNA, which could not have the effect on miR-1307. Moreover, as shown in Fig. 5b, the ING5 expression was increased following the loss of miR-1307 in the ASO-1307 or in the siRNA con + miR-1307 ASO group (*P* < 0.05, respectively), but the ING5 expression was not increased by adding ING5 siRNA following the loss of miR-1307 in A2780 or SKOV3 cells (*P* < 0.05, respectively). The results indicated that miR-1307 directly targeted ING5 in ovarian cancer cells. Moreover, the results of annexin V-FITC immunofluorescence assay or IC50 showed that the apoptosis rate or the chemoresistence was increased or decreased following the loss of miR-1307 in the ASO-1307 or in the siRNA con + miR-1307 ASO group in A2780 or SKOV3 cells (*P* < 0.05, respectively, Fig. 5c and d), but the increased cells apoptosis rate or the decreased chemoresistance following the loss of miR-1307 could be reversed by the down-regulation of ING5 in A2780 or SKOV3 cells (*P* < 0.05, respectively, Fig. 5c and D). In summary, these results suggested that miR-1307 could promote the chemoresistance development of ovarian cancer by targeting ING5 in vitro.

### Inhibiting effect of miR-1307 ASO and/or taxol by increasing the ING5 expression against ovarian cancer in vivo

**Fig. 6:**
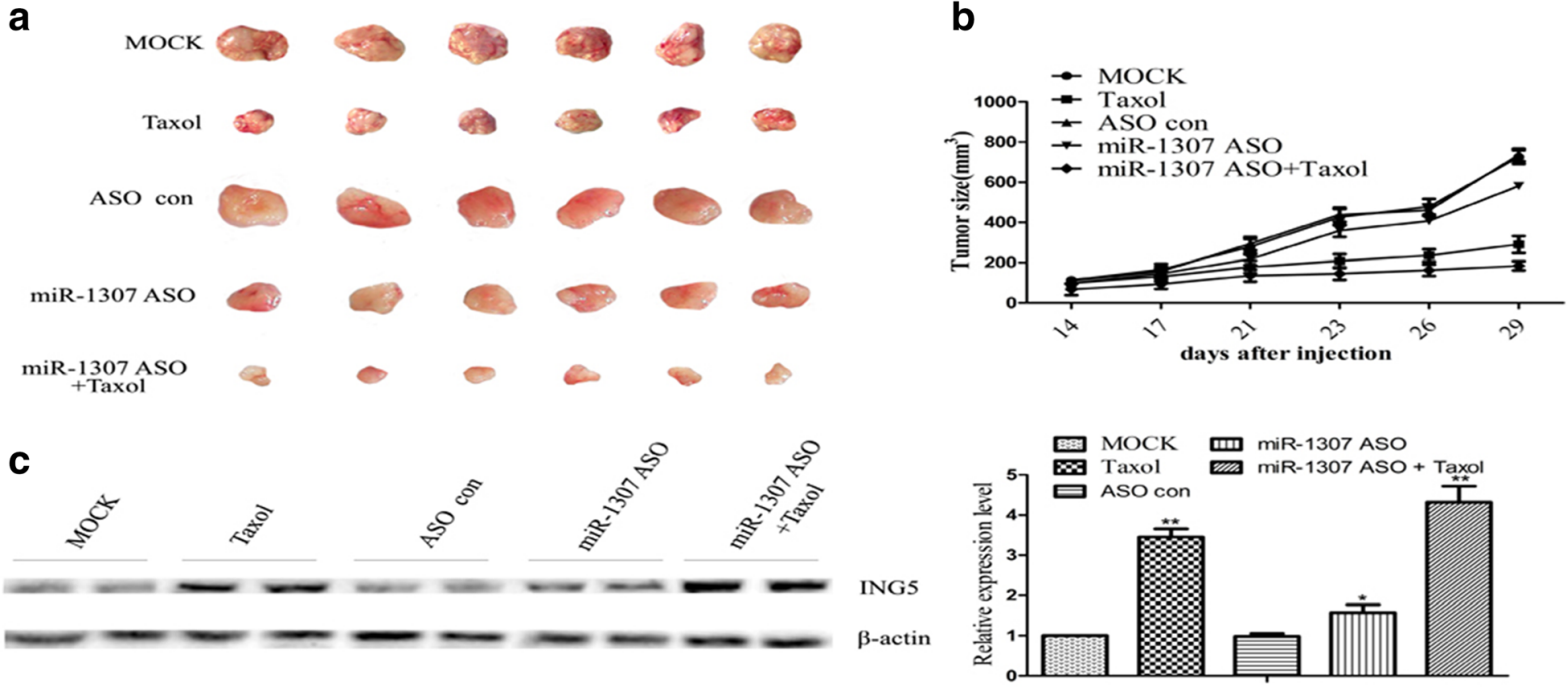
Inhibiting effect of miR-1307 ASO and Taxol by increasing ING5 in vivo. **a** and **b** The mice tumors volume in different groups. After 29 days of treatment, the miR-1307 ASO and/or Taxol therapy decreased the tumors volume compared to the MOCK group, and compared to the ASO con group, the average tumor volume in miR-1307 ASO and miR-1307 ASO + Taxol group was decreased significantly (*P* < 0.05 and *P* < 0.01, respectively). Besides, compared to the Taxol group, the average tumor volume in miR-1307 ASO + Taxol group was decreased significantly (*P* < 0.05). **c** Western Blot results of ING5 expression in different groups. ING5 expression was increased in the miR-1307 ASO and/or Taxol treated group in ovarian cancer cells injected tumor xenografts in nude mice, and its expression was higher in the miR-1307 ASO + Taxol treated group than that in the miR-1307 ASO treated group (*P* < 0.01). Results are representative of at least three separate experiments. Data are expressed as the mean ± standard deviation. Data from three experimental determinations and bars indicate the SD (*P < 0.05, **P < 0.01)

To validate the inhibiting effect of miR-1307 ASO and/or Taxol and to determine whether miR-1307 regulate ING5 expression in vivo, we injected A2780 cells subcutaneously into the right flank of nude mice. The results were as follows: compared to the MOCK group, the average tumor volume in Taxol group was decreased significantly (*P* < 0.01, Fig. 6a and b). And compared to the ASO con group, the average tumor volume in miR-1307 ASO and miR-1307 ASO + Taxol group was decreased significantly (*P* < 0.05 and *P* < 0.01, respectively, Fig. 6a and b). Besides, compared to the Taxol group, the average tumor volume in miR-1307 ASO + Taxol group was decreased significantly (*P* < 0.05, Fig. 6a and b). In addition, we evaluated the ING5 expression in the mice tumors by western blot, and the results suggested that expression of ING5 was increased as a result of miR-1307 ASO and/or Taxol (*P* < 0.01, *P* < 0.05 and *P* < 0.01, respectively, Fig. 6c). Moreover, compared to the Taxol group, the expression of ING5 in miR-1307 ASO + Taxol group was increased (Fig. 6c). Treatment with miR-1307 ASO and/or Taxol resulted in no significant difference in the body weight of treated mice, none of the tested mice manifested signs of other adverse effects as specified in the method section, and no toxicity on the blood count or hepatic and renal function was observed (data not shown). These results indicated the anti-tumor effect and safety of miR-1307 ASO and/or Taxol in vivo. To sum up, these results suggested that miR-1307 could promote the development the chemoresistance of ovarian cancer by targeting ING5 in vivo.

## Discussion

MiRNAs play important roles in almost all tumor cellular processes [2, 5–12], which either promote carcinogenesis or suppress tumor development in ovarian cancer [2, 8–12]. Particularly, many miRNAs including miR-1307 have been linked to chemo-resistance to many kinds of chemotherapy drugs in ovarian cancer [2, 4, 7–9, 13–16]. Recently, miR-1307 was found to be up-regulated in a variety of cancers, such as breast cancer, liver cancer, colorectal tumor and ovarian cancer [7, 21–24]. For example, miR-1307-3p was found to be useful for detecting breast cancer in the early stage [21]. And methylated miR-1307 was identified in subjects who went on to develop breast cancer [22]. MiR-1307 in chronic hepatitis C was identified in a recent report [23]. Even a report has indicated that rs7911488 C-allelic pre-miR-1307 binds to MBNL1 and infers with Dicer processing, and then leads to the decreased miR-1307 and increased Bcl-2 expression, thus representing an important process in the initiation of colorectal cancer [24]. Nevertheless, up to now, we have found only one report about the correlation between miR-1307 and the ovarian cancer chemoresistance, which indicated that miR-1307 was associated with the chemoresistance in ovarian cancer [7]. In our article, to demonstrate the function of miR-1307 in chemoresistance and to explore its chemoresistance mechanism, we firstly showed that miR-1307 was over-expressed in chemoresistant ovarian cancer cell line A2780/Taxol, which was consistent with the literature report [7]. And we also found that over-expression or loss of miR-1307 promoted or inhabited chemoresistance in ovarian cancer cells. Besides, to explain the chemoresistance caused by miR-1307, a preliminary analysis was needed, and we found that over-expression of miR-1307 promoted proliferation and inhibited apoptosis. Moreover, we confirmed that the inhibitor of growth 5 (ING5) as a functional target of miR-1307 in ovarian cancer cells. Thus, our discovery demonstrated that miR-1307 might be a tumorous promoter in ovarian cancer cells, and abnormal alteration of miR-1307-ING5 interaction might contribute to the chemoresistant in ovarian cancer.

It is well known that most microRNAs regulate target mRNAs by binding to the 3’ UTR of target genes in a post-transcriptional manner [18, 19]. Therefore, establishing the interrelationship of miRNA and its target genes helps to better understand the molecular mechanism and provide potential therapeutic targets for the clinical treatment of cancers. Although the anti-apoptosis protein Bcl-2, which was a direct target of miR-1307, had been reported to be over-expressed in colorectal cancer [24], we found that ING5 was a target gene of miR-1307 by means of chip analysis and Target Scan software. Meanwhile, the direct targeting was further supported through luciferase reporter assay. As a result, these data suggested that the regulation mechanism of miR-1307 might vary in different kinds of cancers. To confirm the hypothesis that miR-1307 promoted ovarian cancer cell chemoresistance by targeting the ING5 expression, a preliminary analysis was performed, and the regulatory mechanism of miR-1307 and downstream gene ING5 was explored. Recently, a report suggested that miR-193a-3p-regulated ING5 gene activated the DNA damage response pathway and inhibited multi-chemoresistance in bladder cancer [18]. Different from the result owing to the different types of cancer, our results indicated ING5 was targeted by miR-1307, yet we could also use the similar method to prove their correlation [18, 19]. As shown in the present study, we could see the inhibiting effect of miR-1307 ASO by increasing the ING5 expression against chemoresistant ovarian cancer in vitro. Of course, the similar inhibiting effect was demonstrated in vivo. However, it was also needed to demonstrate the effect in other types of animal models before the clinical trial. To further explore the mechanism, we analyzed the protein expressions following different miR-1307 treatment in various kinds of ovarian cancer cells, and the results indicated the downstream gene ING5 of miR-1307 in vitro. Furthermore, the up-regulated ability of cell apoptosis and down-regulated ability of chemoresistance following the loss of miR-1307 was rescued by adding ING5 siRNA in vitro. The methods by analyzing the mechanism were similar with the recent reports [18, 19], and our results indicated that miR-1307 could promote ovarian cancer chemoresistance by targeting ING5.

ING5, one of the ING family genes and as a transcriptional co-activator, has been demonstrated to play important roles in the development of tumor [18, 25–34]. Some studies reported that the decreased ING5 protein and its cytoplasmic translocation were observed in many tumors, including bladder cancer [18], lung cancer [25, 27], oral squamous cell carcinoma [28, 30], head and neck squamous cell carcinoma [31], colorectal [32], pancreatic cancer [29], gastric carcinoma [26, 33] and ameloblastoma [34]. In addition, it was reported that ING5 could affect adhesion, migration, invasion, proliferation and apoptosis of tumor cells [18, 25–34]. Bax and GADD45 were ING5 target apoptotic genes [35, 36]. The anti-proliferative effect of ING5 depended on its interaction with INCA1 [37]. It was worth noting that ING5-mediated chemoresistance was closely related to their apoptotic resistance, Akt activation, and the over-expression of chemoresistance-related genes [18, 26, 30]. For the inhibiting effect on epithelial-mesenchymal transition (EMT), ING5 inhibited lung cancer aggressiveness [27], and ING5 suppressed PI3K/Akt in breast cancer [36]. In spite of these the correlation between ING5 and ovarian cancer still remained unclear. Various studies reported that ING5 expression was down-regulated or ING5 was a negative regulator of chemoresistance in many kinds of tumors [18, 25–34]. Consistent with these results, we demonstrated that ING5 inhibited cell proliferation, promoted cell apoptosis and inhabited chemoresistance in ovarian cancer.

Chemotherapy is a double-edged sword, which leads to the treatment and the chemoresistance for tumor. This provides a theoretical basis for the therapy, which needs chemotherapy combined with the inhibition of miR-1307. Even so, future studies should also determine the ING5 feedback effect (the pathways associated with ING5 in the above) on miR-1307 during the chemoresistance in ovarian cancer cells, and the above mechanism should be further investigated in various kinds of ovarian cancer cells.

## Conclusions

In conclusion, our data suggested that miR-1307 could promote ovarian cancer chemoresistance by targeting the ING5 expression and miR-1307 might serve as a therapeutic target for ovarian cancer.

## Abbreviations

DAPI: 4,6-diamino-2-phenyl indole
EOC: Epithelial ovarian carcinoma
FITC: Fuorescein isothiocyanate
HRP: Horseradish peroxidase
IC50: Half maximal inhibitory concentration
ING5: Inhibitor of growth 5
miRNA: miR, microRNA
MTT: 3-(4,5-dimethyl-2-thiazolyl)-2,5-diphenyl-2-H-tetrazolium bromide
PBS: Phosphate-buffered saline
PCNA: Proliferating cell nuclear antigen
QRT-PCR: Quantificational real-time polymerase chain reaction
